# Platform-based academic interaction and self-regulated learning in a WeChat-centered social media environment: information overload, perceived control, and information anxiety

**DOI:** 10.3389/fpsyg.2026.1893472

**Published:** 2026-09-21

**Authors:** Lingyun Gao, Feng Zhang

**Affiliations:** School of Education, Beijing Institute of Technology, Beijing, China

**Keywords:** information anxiety, information overload, perceived control, platform-based academic interaction, self-regulated learning, social media learning, WeChat

## Abstract

Social media platforms increasingly support course communication, peer collaboration, and academic information exchange within university learning. In China, these activities are frequently embedded in WeChat-centered communication streams characterized by persistent connectivity, fragmented information sharing, and overlap between academic and personal communication. Drawing on cognitive load theory, control-value theory, and social cognitive theory, this study examined how platform-based academic interaction was associated with self-regulated learning through information overload, perceived control, and information anxiety. Survey data were collected from 846 Chinese university students, of whom 98.5% reported using WeChat for academic purposes and 91.7% identified WeChat as their primary academic communication platform. Structural equation modeling indicated a positive direct association between platform-based academic interaction and self-regulated learning, while the estimated indirect associations involving information overload, perceived control, and information anxiety were negative and larger in absolute magnitude, yielding a negative estimated total association. Higher perceived information literacy was associated with a weaker positive relationship between information overload and information anxiety, whereas higher learning self-efficacy was associated with a weaker negative relationship between information anxiety and self-regulated learning. The information overload to information anxiety association differed across academic stages and was strongest among master’s students. These findings identify information-processing demand, regulatory appraisal, and information-related anxiety as distinct processes associated with self-regulated learning in a WeChat-centered social media environment. The cross-sectional self-report design limits temporal and causal inference.

## 1 Introduction

### 1.1 Social media learning in a WeChat-centered communication environment

University learning increasingly extends beyond formal classrooms and learning-management systems into social media and digital communication spaces. Students receive course requirements, exchange resources, coordinate group tasks, communicate with instructors and peers, and follow academic opportunities through platforms that were not designed exclusively for teaching. Social media has consequently been conceptualized as a learning space in which formal educational activity intersects with communication and participation across settings with varying degrees of formality (Greenhow and Lewin, 2016; Greenhow and Galvin, 2020; Manca, 2020). These environments may increase access to information and social support while also embedding learning within rapid, socially visible, and continuously updated communication streams.

In China, WeChat has become an important part of this learning environment. Its group communication functions allow teachers and students to circulate notices, files, links, screenshots, reminders, questions, and informal explanations within the same platform. Recent qualitative research has shown that WeChat groups involving instructors and groups organized independently by students can support resource sharing, peer assistance, and collaborative learning. The same environments may also generate information overload, fragmented communication, off-topic exchanges, unequal participation, and expectations of continuous instructor availability (Tan and Ren, 2025).

Research on online social media and self-regulated learning similarly suggests that social media may sustain motivation, resource seeking, peer interaction, and learning continuity. Its entertaining and socially embedded features may also introduce distraction, unreliable information, and anxiety. Cai and Yang (2025), for example, found that social media resources and peer interaction could support motivation regulation across phases of self-regulated learning, while learners also needed to manage distracting or unreliable content.

These findings portray social media as a learning environment whose consequences are shaped by the organization and regulation of academic interaction and information. Recent quantitative research has further shown that information and communication technology use is associated with self-regulated learning through information retrieval and social interaction (Wang M. et al., 2025), highlighting the enabling role of digital academic interaction. Current research has nevertheless offered limited insight into the concurrent processes through which platform-based interaction provides learning resources while also increasing the difficulty of managing academic information. Examining these processes can clarify how platform-based academic interaction is associated with self-regulated learning under conditions of increasing information pressure.

The present study examines these processes in a WeChat-centered social media learning environment. Platform-based academic interaction refers to students’ use of digital communication platforms for course communication, peer collaboration, and academic information acquisition, consistent with research describing social media as a setting for communication, participation, resource exchange, and learning across formal and informal educational contexts (Greenhow and Lewin, 2016; Greenhow and Galvin, 2020; Manca, 2020). Academic information pressure provides an organizing concept for the contextual demands surrounding platform-based learning. It encompasses demands arising when the volume, speed, fragmentation, ambiguity, and social immediacy of academic information increase the effort required to manage information effectively, drawing on research concerning information overload and information-related anxiety in complex information environments (Eppler and Mengis, 2004; Bawden and Robinson, 2009).

### 1.2 Self-regulated learning and competing associations of platform interaction

Self-regulated learning involves learners’ active regulation of goals, planning, attention, monitoring, effort, and strategy adjustment (Pintrich, 2004; Zimmerman, 2002; Panadero, 2017). These processes are especially important in digital environments because learners must organize academic activity across multiple communication channels, shifting task demands, and frequent interruptions.

Within social media learning environments, these established regulatory processes operate alongside continuing demands to identify relevant information, coordinate communication, manage interruptions, and integrate information distributed across multiple messages and channels. The manageability of academic information therefore forms an important contextual condition for the enactment of self-regulated learning.

Recent studies continue to show that digital literacy, perceived usefulness, interactive learning environments, self-efficacy, and online learning conditions are associated with students’ capacity to regulate learning (Chen, 2025; Liu et al., 2025). Chen (2025), for example, reported relationships among digital literacy, learning adaptation, online self-regulated learning, and academic achievement. Liu et al. (2025) similarly identified perceived usefulness and interactive learning environments as predictors of self-regulation in generative AI assisted learning.

Platform-based academic interaction may support self-regulated learning by helping students clarify task requirements, obtain learning materials, coordinate group work, receive feedback, and identify academic opportunities. Social interaction may also facilitate help seeking and provide external reminders that support planning and monitoring.

The same interaction may create disruptive processes. Students may need to distinguish relevant from irrelevant messages, integrate fragmented information, verify uncertain claims, monitor multiple groups, and reconstruct task requirements from informal exchanges. These activities consume time and cognitive resources that might otherwise be used for sustained attention, comprehension monitoring, and strategy adjustment.

Recent research with university students has linked information overload and social overload with social media fatigue, cognitive depletion, and lower academic performance. Self-control also appears to reduce some of the adverse associations arising from overload (Wang X. et al., 2025).

The relationship between platform interaction and self-regulated learning may therefore contain statistical components operating in different directions. The term competing associations describes the coexistence of a positive direct association with self-regulated learning and negative indirect associations involving information-related demands, appraisals, and emotional responses. This configuration provides a basis for examining the enabling and demanding components of platform-based academic interaction within the same model.

*H1a:* Platform-based academic interaction is positively associated with information overload.

*H1b:* Platform-based academic interaction is negatively associated with perceived control.

*H1c:* Platform-based academic interaction is positively associated with information anxiety.

*H1d:* Platform-based academic interaction is positively associated with self-regulated learning after information overload, perceived control, and information anxiety are considered.

### 1.3 Information overload, perceived control, and information anxiety

Information overload, perceived control, and information anxiety occupy different theoretical positions within the proposed model. Information overload represents an information-processing demand associated with the amount and manageability of available information (Eppler and Mengis, 2004). Perceived control represents a regulatory appraisal concerning whether academic information demands can be managed effectively (Skinner, 1996; Pekrun, 2006). Information anxiety represents a cognitive and emotional response involving worry, uncertainty, and tension associated with difficulties in managing needed information (Bawden and Robinson, 2009). Their theoretical differentiation organizes the model around informational demand, appraisal of manageability, and information-related cognitive and emotional response.

#### 1.3.1 Information overload

Information overload occurs when information demands exceed an individual’s perceived capacity to attend to, process, organize, or act on available information (Eppler and Mengis, 2004; Karr-Wisniewski and Lu, 2010). In a social media learning environment, overload can arise through the quantity of messages, the speed of updating, fragmented presentation, duplication, unclear priorities, and the effort required to separate academic information from unrelated communication.

Cognitive load theory provides a resource-based account of how these broader informatio’n demands become consequential for learning. Working-memory resources are limited, and the processing required to manage message volume, redundancy, fragmentation, filtering, and information integration consumes resources needed for task-relevant cognitive activity (Paas et al., 2003; Sweller et al., 2019; Sweller, 2020). Information overload in the present study captures this broader information-processing burden. Transitions between platform communication and learning tasks may add further processing costs because shifts between cognitive tasks are associated with measurable switching costs (Monsell, 2003). These demands may reduce the resources available for planning, attention regulation, monitoring, and strategy adjustment.

Information overload may also be associated with learning through students’ appraisal of whether the surrounding information environment remains manageable. When information is perceived as excessive or poorly organized, students may experience lower control over task priorities, study time, and interruptions.

*H2a:* Information overload is negatively associated with perceived control.

Information overload may additionally be associated with greater uncertainty and psychological tension. Students may worry that they have overlooked relevant information, misunderstood a message, or failed to process information in time.

*H2b:* Information overload is positively associated with information anxiety.

Information overload may also be associated directly with lower self-regulated learning because attention and time are diverted toward message monitoring, filtering, and integration.

*H2c:* Information overload is negatively associated with self-regulated learning.

#### 1.3.2 Perceived control

Perceived control concerns students’ appraisal of whether academic information demands remain manageable, consistent with broader conceptualizations of perceived control as judgments concerning the manageability of relevant events and demands (Skinner, 1996). In the present context, it includes perceived control over when information is processed, whether irrelevant messages interrupt learning, how priorities are identified, and whether students can maintain their own learning rhythm.

Control-value theory identifies perceived control as an important antecedent of academic emotions and learning behavior (Pekrun, 2006; Pekrun et al., 2017). When students perceive learning demands as manageable, they are better positioned to sustain effort and use adaptive strategies. When the information environment is experienced as unpredictable or difficult to govern, anxiety and behavioral disruption become more likely.

Recent latent variable research continues to indicate close relationships among regulatory processes, achievement emotions, and academic engagement, while also recognizing that these relationships may be reciprocal. Shi and Li (2026), for example, found that self-regulated learning was associated with academic engagement partly through positive and negative achievement emotions.

Perceived control is conceptually distinct from learning self-efficacy. Learning self-efficacy concerns confidence in one’s ability to carry out learning activities and persist through difficulty (Zimmerman, 2000). Perceived control in this study refers more specifically to the manageability of the information environment surrounding learning.

*H3a:* Perceived control is negatively associated with information anxiety.

*H3b:* Perceived control is positively associated with self-regulated learning.

#### 1.3.3 Information anxiety

Information anxiety refers to cognitive and emotional tension associated with difficulty locating, evaluating, processing, securing, organizing, or keeping up with information needed for academic tasks (Bawden and Robinson, 2009). It is related to information overload but represents a different functional level. Information overload describes a perceived demand condition, whereas information anxiety describes the worry, uncertainty, and unease associated with difficulty governing that condition.

The items developed for the present study covered six content domains: concerns about information retrieval, concerns about information quality, concerns about processing, concerns about security, dependence on platform communication, and concerns about missing important updates. These domains were designed to broaden content coverage, with their empirical dimensionality evaluated separately in the measurement analysis.

Research on fear of missing out, online social anxiety, and media multitasking indicates that uncertainty about digital communication and missed information can become psychologically consequential for university students (Wu et al., 2025).

Information anxiety may interfere with self-regulated learning by diverting attention toward monitoring and uncertainty. It may also make it more difficult for students to maintain plans, return to tasks after interruption, and judge whether their strategies remain effective.

*H3c:* Information anxiety is negatively associated with self-regulated learning.

### 1.4 Conditional roles of perceived information literacy and learning self-efficacy

Students differ in the resources they can use to manage platform-based academic information. This study focuses on perceived information literacy and learning self-efficacy because they are expected to moderate different associations within the proposed model.

Perceived information literacy refers to students’ self-assessed capacity to evaluate information quality, compare sources, identify task-relevant content, organize academic materials, and verify information before using it. Research on information-literacy self-efficacy conceptualizes such judgments as self-assessments of capability in locating, evaluating, organizing, and using information (Kurbanoglu et al., 2006; Aharony and Gazit, 2020; Shonfeld et al., 2022).

Recent higher-education research further indicates that digital literacy is associated with learning adaptation, digital learning, self-efficacy, online self-regulated learning, and learning outcomes (Chen, 2025; Yuan et al., 2025).

Students with stronger perceived information literacy may be better able to distinguish relevant from irrelevant messages, evaluate credibility, reduce uncertainty, and organize fragmented information into usable learning resources.

*H4a:* Perceived information literacy moderates the association between information overload and information anxiety, such that the positive association is weaker at higher levels of perceived information literacy.

Learning self-efficacy refers to students’ confidence in managing learning challenges, persisting through difficulty, and recovering from disruption. Self-efficacy beliefs influence effort, persistence, strategy use, and responses to obstacles (Bandura, 1977; Zimmerman, 2000; Schunk and DiBenedetto, 2020).

Recent research in digital and blended learning continues to identify self-efficacy as an important correlate of metacognitive strategy use, self-regulated learning, and perceived academic performance. Xie (2026) similarly found that self-efficacy was closely connected with metacognitive strategies in blended science learning.

Learning self-efficacy may help students maintain effort, re-establish goals, and continue using learning strategies after anxiety has emerged.

*H4b:* Learning self-efficacy moderates the association between information anxiety and self-regulated learning, such that the negative association is weaker at higher levels of learning self-efficacy.

### 1.5 Academic-stage heterogeneity

Undergraduate, master’s, and doctoral students face different configurations of coursework, research, assessment, career preparation, and academic communication. Because prior evidence was insufficient to formulate directional hypotheses for all stage comparisons, academic-stage differences were treated as exploratory.

*RQ1:* Do the paths from information overload to information anxiety, from information anxiety to self-regulated learning, and from perceived control to self-regulated learning differ across undergraduate, master’s, and doctoral students?

### 1.6 The present study and hypothesized model

The present study develops an integrated model to explain how platform-based academic interaction is associated with self-regulated learning in a WeChat-centered social media learning environment. The model brings together the enabling function of platform interaction and the information-related demands that may accompany such interaction.

As shown in Figure 1, platform-based academic interaction is specified as the focal predictor. Information overload, perceived control, and information anxiety represent information-processing demand, regulatory appraisal, and cognitive and emotional response, respectively. Self-regulated learning is specified as the focal learning outcome.

**Figure 1:**
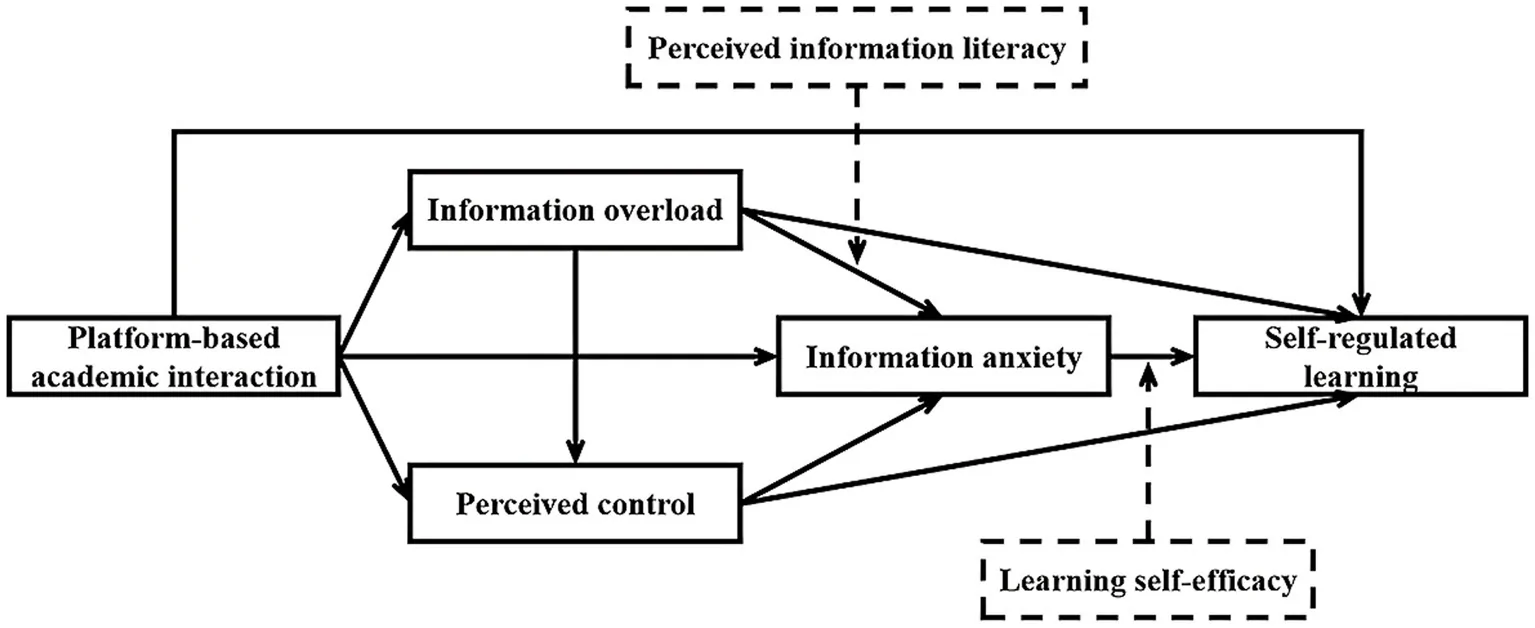
Hypothesized model of competing associations in a WeChat-centered social media learning environment. Solid arrows represent hypothesized structural associations. Dashed arrows point to the paths moderated by perceived information literacy and learning self-efficacy. Academic stage is examined through exploratory multigroup comparisons of the path from information overload to information anxiety, the path from information anxiety to self-regulated learning, and the path from perceived control to self-regulated learning.

The model includes direct paths from platform-based academic interaction to information overload, perceived control, information anxiety, and self-regulated learning. It also includes paths from information overload to perceived control, information anxiety, and self-regulated learning; paths from perceived control to information anxiety and self-regulated learning; and a path from information anxiety to self-regulated learning.

The structural specification follows the functional distinctions developed above. The direct path from platform-based academic interaction to self-regulated learning captures the enabling component associated with access to academic resources, coordination, feedback, and help seeking. The paths involving information overload, perceived control, and information anxiety capture information-management processes involving processing demand, appraisal of manageability, and cognitive and emotional response. The direct paths from platform-based academic interaction to perceived control and information anxiety allow the model to assess whether platform interaction is associated with manageability appraisals and information-related tension beyond the informational burden represented by overload. Such associations are theoretically plausible because persistent platform communication can involve interruption, immediacy, social visibility, and uncertainty about whether important information has been missed (Bawden and Robinson, 2009; Tan and Ren, 2025).

The model further proposes that perceived information literacy moderates the path from information overload to information anxiety, whereas learning self-efficacy moderates the path from information anxiety to self-regulated learning. Academic stage is examined through exploratory multigroup comparisons.

The positions of the two moderators correspond to their conceptual functions. Perceived information literacy concerns students’ capability to evaluate, verify, select, and organize information and is therefore examined in relation to the association between information overload and information anxiety. Learning self-efficacy concerns students’ confidence in sustaining learning activity when difficulties arise and is therefore examined in relation to the association between information anxiety and self-regulated learning.

The study contributes in three respects. First, it examines enabling and demanding components of platform-based academic interaction within the same model. Second, it differentiates information-processing demand, regulatory appraisal, and information-related cognitive and emotional response within the management of academic information. Third, it examines whether two forms of perceived learner capability are associated with variation in different relationships within the proposed model. The structural specification shown in Figure 1 was fixed *a priori* on the basis of the theoretical relationships developed above. The prespecified model was estimated without adding structural paths or correlated residuals on the basis of modification indices. Alternative-model comparisons served as secondary specification checks.

The ordering of the variables in Figure 1 is theoretically informed and provides the directional structure for hypothesis testing. The cross-sectional design limits inference concerning temporal ordering and causality.

## 2 Materials and methods

### 2.1 Research design

The study used a cross-sectional questionnaire design. The theoretical model ordered platform-based academic interaction, information overload, perceived control, information anxiety, and self-regulated learning according to cognitive load theory, control-value theory, and social cognitive theory. All variables were measured at one time point, and the structural paths are interpreted as theoretically ordered statistical associations.

All main constructs were assessed using five-point Likert-type items ranging from 1 (strongly disagree), to 5 (strongly agree).

### 2.2 Participants and procedure

Eligible participants were full-time undergraduate, master’s, and doctoral students enrolled at universities in China who had experience using digital communication platforms for academic communication, learning coordination, peer collaboration, or academic information acquisition. Participants were recruited from six universities located in eastern, central, and western China. The participating institutions included comprehensive universities, science and engineering universities, and teacher education universities. Recruitment followed a non-probability, multi-institutional convenience sampling strategy. The inclusion of universities from different regions and institutional types increased heterogeneity in the analytical sample, while the sampling design limits population-level representativeness. Data were collected between January and May 2026 using Wenjuanxing (Questionnaire Star), a Chinese online survey platform. The questionnaire link was distributed through course communication channels and student group networks. To avoid placing disproportionate emphasis on anxiety before participation, the recruitment materials described the project as a study of academic information and learning experiences.

Participation was voluntary and anonymous. Before beginning the questionnaire, participants received information about the purpose and procedures of the study, the voluntary nature of participation, the confidentiality of their responses, the intended use of the data, and their right to discontinue participation. Only participants who provided informed consent proceeded to the questionnaire. No directly identifying personal information was collected.

Before the main survey, a pilot study involving 62 university students was conducted to examine item clarity, contextual appropriateness, and the overall flow of the questionnaire. Feedback from the pilot study was used to clarify the meaning of several items and improve their alignment with the platform-based academic communication context. These revisions focused on semantic refinement, while the number of items and the substantive content covered by the measures remained unchanged.

The main survey yielded 912 responses. Two reverse-worded items were embedded in the questionnaire as response-quality checks. Responses were screened according to predetermined data-quality criteria and were excluded when the answers to these items indicated insufficient attentiveness, when the questionnaire was completed in an implausibly short period, or when invariant or clearly patterned responding was observed across extended item blocks. After screening, 846 responses were retained for analysis, corresponding to a retention rate of 92.8%.

The analytical sample comprised 386 male students and 460 female students. It included 331 undergraduate students, 362 master’s students, and 153 doctoral students. Participants represented humanities, social sciences, education, natural sciences, engineering, medicine, and interdisciplinary fields. Detailed participant and platform-use characteristics are presented in Table 1. The adequacy of the sample was considered in relation to the complexity of the measurement and structural models, the number and magnitude of the factor loadings, and the demands of maximum-likelihood estimation, consistent with simulation-based recommendations for SEM sample-size evaluation (Wolf et al., 2013). Because the doctoral subgroup was smaller than the undergraduate and master’s subgroups, the multigroup findings were interpreted cautiously.

**Table 1:** Participant and social media context characteristics.

Characteristic	Category	n	%
(A) Participant characteristics.			
Gender	Male	386	45.6
	Female	460	54.4
Academic stage	Undergraduate	331	39.1
	Master’s	362	42.8
	Doctoral	153	18.1
Discipline	Humanities	124	14.7
	Social sciences	183	21.6
	Education	106	12.5
	Natural sciences	132	15.6
	Engineering	201	23.8
	Medicine	64	7.6
	Interdisciplinary/other	36	4.3
Daily platform learning use	Less than 30 min	74	8.7
	30–60 min	168	19.9
	1–2 h	287	33.9
	2–3 h	209	24.7
	More than 3 h	108	12.8

Indicator	Category	n	%
(B) Platform context			
Primary academic platform	WeChat	776	91.7
	QQ	37	4.4
	DingTalk	18	2.1
	LMS/other	15	1.8
WeChat academic use	Yes	833	98.5
	No	13	1.5

Context indicator	M	SD	Responses of 4 or 5 (%)
(C) Social media context indicators			
Continuous connectivity	3.79	0.846	64.3
Academic–personal boundary blurring	3.534	0.939	51.4
Fragmented information sharing	3.831	0.856	65.6
Responsiveness expectations	3.68	0.889	59.2
Information-quality concerns	3.382	0.854	45.6
Missing-update pressure	3.755	0.912	62.8

The six context indicators were analyzed separately as descriptive indicators of the communication context.

### 2.3 WeChat-centered social media learning context

The research context was characterized as WeChat-centered on the basis of participants’ platform-use responses. Of the 846 cases, 776 respondents identified WeChat as their primary platform for academic communication, and 833 reported using WeChat for academic communication or learning. QQ, DingTalk, and learning-management or other platforms were used as the primary platform by a smaller proportion of respondents.

The substantive scales assessed academic interaction across digital communication platforms. Accordingly, the term WeChat-centered social media learning environment describes the dominant communication context of the sample, while the measured construct captures platform-based academic interaction across the communication channels used by participants.

Six single-item context indicators described continuous connectivity, overlap between academic and personal communication, fragmented information sharing, expectations of timely responsiveness, concern about information quality, and pressure associated with missing important updates. These indicators were reported separately as heterogeneous descriptive properties of the communication context.

Participant characteristics, platform-use patterns, and the social media context indicators are presented in Table 1.

### 2.4 Measures

The survey was administered in Chinese. All substantive construct items were rated on a five-point Likert scale ranging from 1 (strongly disagree) to 5 (strongly agree), with higher scores indicating higher levels of the corresponding constructs. Two reverse-worded items were included solely as response-quality checks and were not included in the scoring or measurement analysis of any substantive construct. Complete English item wording of the substantive construct items is provided in Supplementary Table S1. The English wording was prepared for reporting purposes from the Chinese questionnaire administered to participants.

Construct definitions and relevant item content were identified from peer-reviewed English-language research and contextually adapted to Chinese university students’ platform-based academic communication and information-management experiences. The initial Chinese item pool underwent qualitative review by an expert in educational psychology, focusing on conceptual relevance, wording clarity, contextual appropriateness, and differentiation among adjacent constructs. A pilot study involving 62 university students then examined item comprehensibility and survey flow. Feedback from these two stages led to semantic refinement of several items, particularly where wording could blur the distinctions among information overload, perceived control, information anxiety, and perceived information literacy. The planned number of items and substantive content coverage remained unchanged.

Because several measures were contextually adapted or developed for the present study, their internal consistency and measurement properties were evaluated before the structural associations were examined. Confirmatory factor analysis was used to assess standardized factor loadings and the empirical distinctiveness of the constructs. Reliability was evaluated using Cronbach’s alpha and composite reliability, convergent validity was examined using average variance extracted, and discriminant validity was assessed using heterotrait-monotrait ratios.

#### 2.4.1 Platform-based academic interaction

Platform-based academic interaction was measured with 12 items covering course communication, peer collaboration, and academic information acquisition. Example items included “I use digital platforms to discuss academic tasks with classmates or peers” and “Digital platforms are an important channel for acquiring academic information in my daily learning.” The items were informed by peer-reviewed research on social media supported learning, digital academic communication, and social media as a higher-education learning environment (Greenhow and Galvin, 2020; Manca, 2020; Barrot, 2022).

#### 2.4.2 Information overload

Information overload was measured with six items covering information quantity, speed, fragmentation, filtering difficulty, and redundancy. An example item was “Academic information on platforms is often fragmented and difficult to integrate.” The items were informed by peer-reviewed conceptual and empirical research on information overload and technology overload (Eppler and Mengis, 2004; Karr-Wisniewski and Lu, 2010; Pang, 2021).

#### 2.4.3 Perceived control

Perceived control was measured with five items assessing timing control, interruption control, perceived manageability, learning rhythm, and overall control of platform-based academic information. An example item was “I can maintain my own learning rhythm despite frequent academic messages.” The items were informed by peer-reviewed research on control appraisals and achievement emotions (Skinner, 1996; Pekrun, 2006).

#### 2.4.4 Information anxiety

Information anxiety was measured using 18 items covering six theoretically informed content domains: information retrieval, information quality, information processing, information security, dependence on platform communication, and missing important academic updates. Example items included “I worry that I cannot find important learning information when I need it,” “I feel anxious when different platform messages provide inconsistent academic information,” and “I worry that others may receive important academic updates earlier than I do.” The items were developed with reference to peer-reviewed research on information anxiety, information overload, fear of missing out, and dependence on digital communication (Bawden and Robinson, 2009; Dhir et al., 2018; Elhai et al., 2020; Wu et al., 2025).

The six content domains were used to ensure breadth of content coverage. Their empirical dimensionality was evaluated using correlated six-factor and second-order specifications, both of which produced inadmissible parameter estimates. The 18 items were modeled as indicators of a single information-anxiety factor, which provided the admissible measurement representation used in the primary structural analysis. Its dimensional interpretation is treated as provisional pending independent validation.

#### 2.4.5 Self-regulated learning

Self-regulated learning was measured with 12 items covering planning, attention regulation, monitoring, and strategy adjustment. Example items included “I arrange my study time according to task priorities” and “I adjust my learning strategies when necessary.” The item pool was informed by peer-reviewed research on self-regulated learning in online and blended environments (Barnard et al., 2009; Broadbent and Poon, 2015; Panadero, 2017).

#### 2.4.6 Perceived information literacy

Perceived information literacy was measured with five items assessing information evaluation, source comparison, relevance judgment, information organization, and information verification. Item development was informed by peer-reviewed research on information-literacy self-efficacy and individuals’ perceived capacity to evaluate and manage information (Kurbanoglu et al., 2006; Aharony and Gazit, 2020; Shonfeld et al., 2022).

Example items included “I can judge whether platform-based academic information is reliable,” “I can identify which platform messages are relevant to my learning goals,” and “I can verify important learning information before acting on it.”

The items elicit respondents’ judgments of their own information-related capabilities, consistent with the self-efficacy orientation of the source literature (Kurbanoglu et al., 2006; Aharony and Gazit, 2020). The construct is therefore referred to as perceived information literacy.

#### 2.4.7 Learning self-efficacy

Learning self-efficacy was measured with five items covering confidence in completing difficult learning tasks, persistence when encountering obstacles, recovery after disruption, management of academic challenges, and continued effort under stress. The items were informed by peer-reviewed research identifying self-efficacy as an important correlate of motivation, persistence, strategy use, and self-regulated learning (Bandura, 1977; Zimmerman and Bandura, 1994; Zimmerman, 2000; Honicke and Broadbent, 2016; Schunk and DiBenedetto, 2020). An example item was “Even when I feel stressed, I can continue working toward my learning goals.”

### 2.5 Data analysis

Analyses were conducted in SPSS Statistics, IBM SPSS Amos, and Python.

SPSS was used for data screening, descriptive statistics, Cronbach’s alpha, Pearson correlations, Harman’s single-factor analysis, multicollinearity checks, preliminary regressions based on composite scores, institutional clustering diagnostics, item-level reliability diagnostics, and reduced-item sensitivity analyses.

AMOS was used for confirmatory factor analysis, comparison of alternative information-anxiety measurement models, structural equation modeling, bootstrap estimation of direct, indirect, and total associations between latent variables, competing-model comparison, measurement invariance, and comparisons across academic stages. Maximum-likelihood estimation was used. Bias-corrected 95% bootstrap confidence intervals based on 5,000 resamples were obtained for the structural paths and for the direct, total indirect, and total latent-variable associations reported in Table 2.

**Table 2:** Direct, indirect, and total associations.

Predictor and outcome	Direct β	95% BC CI	Total indirect β	95% BC CI	Total β	95% BC CI
(A) Selected latent-variable direct, total indirect, and total associations estimated in Amos						
PAI and IA	0.172	[0.112, 0.233]	0.289	[0.245, 0.336]	0.461	[0.399, 0.517]
PAI and SRL	0.240	[0.182, 0.298]	−0.382	[−0.446, −0.317]	−0.142	[−0.210, −0.072]
IO and SRL	−0.169	[−0.238, −0.096]	−0.399	[−0.452, −0.349]	−0.568	[−0.633, −0.504]
PC and SRL	0.508	[0.446, 0.568]	0.095	[0.070, 0.124]	0.604	[0.545, 0.659]

Specific indirect path	B	BootSE	95% percentile bootstrap CI	Fully standardized effect
(B) Complementary specific indirect associations estimated from composite scores				
PAI → IO→SRL	−0.082	0.017	[−0.116, −0.050]	−0.080
PAI → PC → SRL	−0.045	0.018	[−0.081, −0.013]	−0.044
PAI → IA → SRL	−0.056	0.011	[−0.079, −0.036]	−0.054
PAI → IO→PC → SRL	−0.105	0.012	[−0.129, −0.083]	−0.102
PAI → IO→IA → SRL	−0.052	0.008	[−0.069, −0.038]	−0.051
PAI → PC → IA → SRL	−0.009	0.004	[−0.017, −0.003]	−0.009
PAI → IO→PC → IA → SRL	−0.021	0.004	[−0.029, −0.015]	−0.021
Total indirect	−0.371	0.032	[−0.436, −0.310]	−0.360
Direct	0.23	0.029	[0.173, 0.287]	0.223
Total	−0.141	0.035	[−0.211, −0.071]	−0.137

Panel A reports selected standardized latent-variable direct, total indirect, and total associations estimated in Amos, with 95% bias-corrected bootstrap confidence intervals based on 5,000 resamples. Panel B reports ordinary least squares estimates based on composite scale scores. Within Panel B, the seven specific indirect estimates sum, subject to rounding, to the composite-score total indirect association. The latent-variable total indirect associations in Panel A are estimated separately. Bootstrap intervals in Panel B are percentile intervals based on 5,000 samples.

Model evaluation considered the chi-square statistic, chi-square divided by degrees of freedom, comparative fit index, Tucker-Lewis index, root mean square error of approximation, and the unstandardized root mean square residual reported by Amos. The indices were interpreted jointly in accordance with recommendations to evaluate multiple complementary fit criteria (Hu and Bentler, 1999; Marsh et al., 2004). The residual index reported by Amos is RMR.

Reliability was evaluated using Cronbach’s alpha and composite reliability. Convergent validity was evaluated using average variance extracted (Fornell and Larcker, 1981), and discriminant validity was evaluated using heterotrait-monotrait ratios (Henseler et al., 2015).

Specific indirect associations and moderation models were estimated in Python using composite scale scores. Seven theoretically specified indirect associations were calculated using ordinary least squares path analysis with 5,000 percentile bootstrap samples. Moderation analyses used mean-centered predictors, HC3 robust standard errors, 5,000 bootstrap samples for the interaction coefficients, simple slopes at the moderator mean and one standard deviation below and above the mean, and the Johnson and Neyman procedure (Hayes and Matthes, 2009).

AMOS coefficients represent estimates between latent variables, whereas the Python analyses use composite scale scores. The latent-variable analyses characterize the overall structural relationships among the constructs, while the composite-score analyses provide supplementary estimates of the specified indirect sequences and moderation relationships. The two sets of estimates are interpreted at their respective levels of analysis.

For comparison across academic stages, configural and metric invariance were evaluated before equality constraints were imposed on the path from information overload to information anxiety, the path from information anxiety to self-regulated learning, and the path from perceived control to self-regulated learning.

Sensitivity analyses re-estimated the path equations based on composite scores after controlling for demographic and institutional variables. The analyses were also repeated in the subsample reporting academic WeChat use and the subsample identifying WeChat as the primary academic platform.

Potential institutional clustering was additionally examined using empty random-intercept models for the principal composite scores, with institution specified as the clustering unit. Intraclass correlations were calculated from the estimated between-institution and residual variances. Because the analysis involved six institutions, the ICC estimates were used as descriptive diagnostics of institution-level variance.

The high internal consistency of the 18-item information-anxiety measure also prompted supplementary item-level diagnostics using corrected item-total correlations and alpha-if-item-deleted statistics. Two domain-balanced composite scores containing six and twelve information-anxiety items were then examined as sensitivity specifications. Each reduced specification retained representation of the six content domains, allowing the stability of the principal observed-score associations to be assessed across alternative item sets.

## 3 Results

### 3.1 Descriptive statistics and correlations

Descriptive statistics, internal-consistency estimates, and zero-order correlations are presented in Table 3. All seven composite scores showed high internal consistency.

**Table 3:** Descriptive statistics, reliability, and correlations.

Variable	M	SD	α	1	2	3	4	5	6	7
1. PAI	3.844	0.66	0.968	—						
2. IO	3.636	0.699	0.942	0.477***	—					
3. PC	3.191	0.742	0.938	−0.306***	−0.493***	—				
4. IA	3.511	0.664	0.979	0.449***	0.601***	−0.553***	—			
5. SRL	3.476	0.681	0.969	−0.137***	−0.475***	0.656***	−0.563***	—		
6. IL	3.59	0.669	0.923	0.127***	−0.170***	0.380***	−0.165***	0.476***	—	
7. LSE	3.618	0.681	0.929	0.092**	−0.192***	0.418***	−0.226***	0.504***	0.389***	—

*N* = 846. PAI, platform-based academic interaction; IO, information overload; PC, perceived control; IA, information anxiety; SRL, self-regulated learning; IL, perceived information literacy; LSE, learning self-efficacy. **p < 0.01; ***p < 0.001.

Platform-based academic interaction was positively correlated with information overload and information anxiety and negatively correlated with perceived control and self-regulated learning. The negative zero-order correlation between platform-based academic interaction and self-regulated learning represents the bivariate association between the two variables, whereas the positive direct structural association represents their conditional relationship after information overload, perceived control, and information anxiety are included. This difference is consistent with the proposed competing-association configuration.

### 3.2 Measurement model and information-anxiety specification

The seven-construct measurement model, in which information anxiety was represented as a single latent factor, showed close fit to the observed covariance matrix: χ^2^(1869) = 1918.247, *p* = 0.209, χ^2^/df = 1.026, CFI = 0.999, TLI = 0.999, RMSEA = 0.006, 90% CI [0.000, 0.010], and RMR = 0.011.

Standardized item loadings ranged from 0.823 to 0.882. Composite reliability values ranged from 0.923 to 0.979, and AVE values ranged from 0.706 to 0.753. Item-level standardized factor loadings are reported in Supplementary Table S3, together with construct-level reliability and convergent-validity indices.

HTMT ratios ranged from 0.097 to 0.688. The highest value was between perceived control and self-regulated learning and remained below the 0.85 and 0.90 criteria. The full HTMT matrix is reported in Supplementary Table S4.

Three alternative specifications of information anxiety were evaluated. The single-factor model showed close fit and admissible estimates. The correlated six-factor model produced latent correlations exceeding 1.00, and its covariance matrix was not positive definite. The second-order model produced negative residual variances and standardized second-order loadings exceeding 1.00.

The single-factor model was retained as the admissible representation for the present sample, with its dimensional interpretation treated as provisional. Measurement properties and information-anxiety model comparisons are presented in Table 4.

**Table 4:** Measurement properties and information-anxiety model comparison.

Construct	Items	Standardized loading range	α	CR	AVE
(A) Reliability and convergent validity.					
PAI	12	0.836 to 0.855	0.968	0.968	0.716
IO	6	0.835 to 0.867	0.942	0.942	0.73
PC	5	0.862 to 0.882	0.938	0.938	0.753
IA	18	0.835 to 0.863	0.979	0.979	0.717
SRL	12	0.830 to 0.869	0.969	0.969	0.725
IL	5	0.823 to 0.865	0.923	0.923	0.706
LSE	5	0.840 to 0.860	0.929	0.929	0.723

Model	χ^2^	df	χ^2^/df	CFI	TLI	RMSEA	AIC	BIC	Parameter solution
(B) Alternative information-anxiety measurement models									
Single factor	165.579	135	1.227	0.998	0.998	0.016	237.579	408.237	Admissible
Six correlated factors	140.241	120	1.169	0.999	0.998	0.014	242.241	484.008	Inadmissible
Second-order factor	154.009	129	1.194	0.998	0.998	0.015	238.009	437.111	Inadmissible

The six-factor solution produced latent correlations greater than 1.00, and its covariance matrix was not positive definite. The second-order solution produced negative residual variances and standardized loadings greater than 1.00. The single-factor specification provided the admissible representation used in the present analyses, and its dimensional interpretation is treated as provisional.

Supplementary item-level diagnostics provided further information about the 18-item measure. Corrected item-total correlations ranged approximately from 0.825 to 0.854, while alpha-if-item-deleted values remained approximately between 0.977 and 0.978, compared with α = 0.979 for the full scale. The uniformly high item-total correlations and minimal change in alpha after item deletion indicate substantial commonality across the item pool and suggest considerable similarity among some items. Taken together, the close global fit, narrow loading ranges, and item-level diagnostics indicate a highly homogeneous measurement pattern in the present sample.

### 3.3 Diagnostic and robustness checks

Harman’s single-factor analysis indicated that the first unrotated factor explained 39.24% of the total item variance. The analysis provides a limited diagnostic of single-factor dominance, while common method variance remains a methodological consideration because the principal constructs were measured through self-report in the same cross-sectional survey.

Variance inflation factors in the main equations based on composite scores ranged from approximately 1.00 to 1.92. HC3 robust standard errors produced the same conclusions as ordinary least squares standard errors. Additional diagnostic results are reported in Supplementary Table S5.

The central paths remained similar after gender, academic stage, discipline, daily platform learning use, and institution fixed effects were included. The pattern was also stable among academic WeChat users, *n* = 833, and respondents identifying WeChat as their primary academic platform, *n* = 776. Sensitivity results are reported in Supplementary Table S6.

Empty random-intercept models indicated very small institution-level variance across the principal composite scores, with estimated ICCs ranging from approximately zero to 0.008. The principal composite-score relationships also remained substantively stable after institution indicators were included. These diagnostics indicate limited between-institution variation in the present sample, while the small number of participating institutions constrains institution-level inference.

Reduced-item information-anxiety analyses yielded the same substantive pattern. The six-item domain-balanced score showed α = 0.940 and the twelve-item score showed α = 0.968. Across the two specifications, standardized coefficients ranged from 0.351 to 0.358 for the association between information overload and information anxiety, from −0.306 to −0.319 for the association between perceived control and information anxiety, and from −0.285 to −0.292 for the association between information anxiety and self-regulated learning. The information overload by perceived information literacy interaction ranged from −0.174 to −0.186, and the information anxiety by learning self-efficacy interaction ranged from 0.203 to 0.210. All five associations remained statistically significant at *p* < 0.001. The consistency of these estimates supports the stability of the principal observed-score relationships across alternative representations of the information-anxiety item pool.

### 3.4 Competing structural models

The hypothesized structural model showed a close fit to the observed covariance matrix, χ^2^(1315) = 1345.853, *p* = 0.271, χ^2^/df = 1.023, CFI = 0.999, TLI = 0.999, RMSEA = 0.005, and RMR = 0.011. The alternative specifications were examined as secondary checks of the prespecified structural model. Among the candidate models examined, the retained model yielded the lowest AIC and BIC values, providing relative support for the specified structure. Removing the path from platform-based academic interaction to self-regulated learning significantly worsened model fit. Removing the path from platform-based academic interaction to information anxiety or the path from information overload to self-regulated learning also resulted in poorer fit. The model containing only the direct associations performed substantially worse than the retained model. Fit indices for all candidate models are reported in Table 5.

**Table 5:** Comparison of the hypothesized and alternative structural models.

Model	χ^2^	df	χ^2^/df	CFI	TLI	RMSEA	RMR	AIC	BIC	Δχ^2^
Retained structural model	1345.853	1,315	1.023	0.999	0.999	0.005	0.011	1577.853	2127.754	—
Without PAI → SRL	1410.962	1,316	1.072	0.998	0.998	0.009	0.025	1640.962	2186.122	65.109***
Without PAI → IA	1377.626	1,316	1.047	0.999	0.999	0.007	0.024	1607.626	2152.786	31.773***
Without IO→SRL	1368.111	1,316	1.04	0.999	0.999	0.007	0.014	1598.111	2143.27	22.258***
Direct-path model	2452.212	1,321	1.856	0.975	0.974	0.032	0.135	2672.212	3193.669	1106.359***

Each alternative model is compared with the hypothesized model. ***p < 0.001. Alternative specifications were examined as secondary checks on the theoretically specified structural model.

### 3.5 Structural associations

All ten hypothesized structural paths were statistically significant. Platform-based academic interaction was positively associated with information overload, information anxiety, and self-regulated learning and negatively associated with perceived control.

Information overload was negatively associated with perceived control and self-regulated learning and positively associated with information anxiety. Perceived control was negatively associated with information anxiety and positively associated with self-regulated learning. Information anxiety was negatively associated with self-regulated learning.

The direct association between platform-based academic interaction and perceived control was small, *β* = −0.080, whereas the association between information overload and perceived control was considerably larger, *β* = −0.484. Perceived control had the largest positive association with self-regulated learning, *β* = 0.508.

The final structural model is shown in Figure 2, and detailed estimates are reported in Table 6.

**Figure 2:**
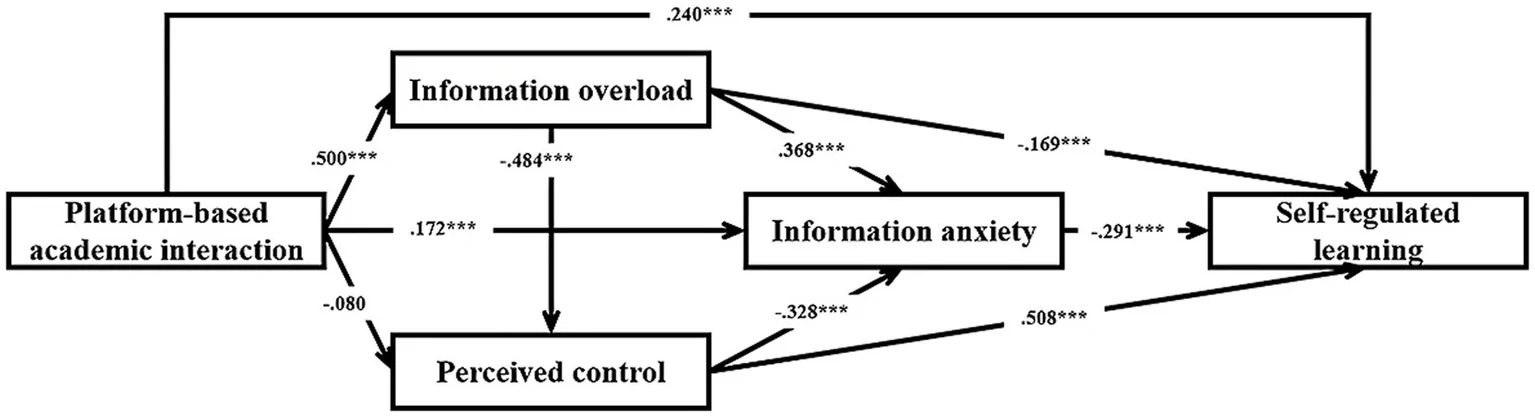
Final structural model with standardized coefficients between latent variables and squared multiple correlations. PAI, Platform-based academic interaction; IO, Information overload; PC, Perceived control; IA, Information anxiety; SRL, Self-regulated learning.

**Table 6:** Structural path estimates and explained variance.

Hypothesis	Path	β	95% BC bootstrap CI	*p*
(A) Structural paths				
H1a	PAI → IO	0.500	[0.440, 0.553]	<0.001
H1b	PAI → PC	−0.080	[−0.149, −0.008]	0.032
H1c	PAI → IA	0.172	[0.112, 0.233]	<0.001
H1d	PAI → SRL	0.240	[0.182, 0.298]	<0.001
H2a	IO→PC	−0.484	[−0.551, −0.421]	<0.001
H2b	IO→IA	0.368	[0.300, 0.433]	<0.001
H2c	IO→SRL	−0.169	[−0.238, −0.096]	<0.001
H3a	PC → IA	−0.328	[−0.386, −0.268]	<0.001
H3b	PC → SRL	0.508	[0.446, 0.568]	<0.001
H3c	IA → SRL	−0.291	[−0.355, −0.224]	<0.001

Endogenous construct	R^2^
(B) Squared multiple correlations	
IO	0.25
PC	0.28
IA	0.498
SRL	0.567

Estimates are standardized coefficients between latent variables from Amos. Confidence intervals are bias-corrected bootstrap intervals based on 5,000 resamples. The p values are maximum-likelihood significance tests for the structural paths.

### 3.6 Direct, indirect, and total associations

The latent-variable model indicated that platform-based academic interaction had a positive direct association with self-regulated learning, *β* = 0.240, 95% bias-corrected bootstrap CI [0.182, 0.298], but a larger negative total indirect association, *β* = −0.382, 95% bias-corrected bootstrap CI [−0.446, −0.317]. The resulting total association was negative, *β* = −0.142, 95% bias-corrected bootstrap CI [−0.210, −0.072].

The direct and indirect components therefore operated in different directions within the estimated overall association. A complementary composite-score analysis estimated seven theoretically specified indirect associations using 5,000 percentile bootstrap samples. All seven specific indirect associations were statistically significant.

The largest specific indirect path extended from platform-based academic interaction to information overload, then to perceived control, and finally to self-regulated learning. Its fully standardized indirect association was −0.102. This indirect path was larger than the path operating only through information overload or any individual path operating through information anxiety.

The latent-variable total indirect association summarizes the overall indirect component of the structural model, while the composite-score estimates describe the relative pattern of the seven specified indirect sequences.

The direct, indirect, and total associations are presented in Table 2.

### 3.7 Moderating roles of perceived information literacy and learning self-efficacy

#### 3.7.1 Perceived information literacy

The interaction between information overload and perceived information literacy was negative and statistically significant, B = −0.251, 95% percentile bootstrap CI [−0.315, −0.190]. Adding the interaction increased explained variance in information anxiety by 0.034.

At one standard deviation below the mean of perceived information literacy, the conditional association between information overload and information anxiety was B = 0.513. At the mean, it was B = 0.345. At one standard deviation above the mean, it was B = 0.178. All three slopes remained statistically significant, while their magnitude decreased as perceived information literacy increased.

The Johnson-Neyman procedure identified an observed-scale threshold of approximately 4.635. Above this value, the positive association between information overload and information anxiety was no longer statistically significant within the observed range.

The conditional pattern is illustrated in Figure 3.

**Figure 3:**
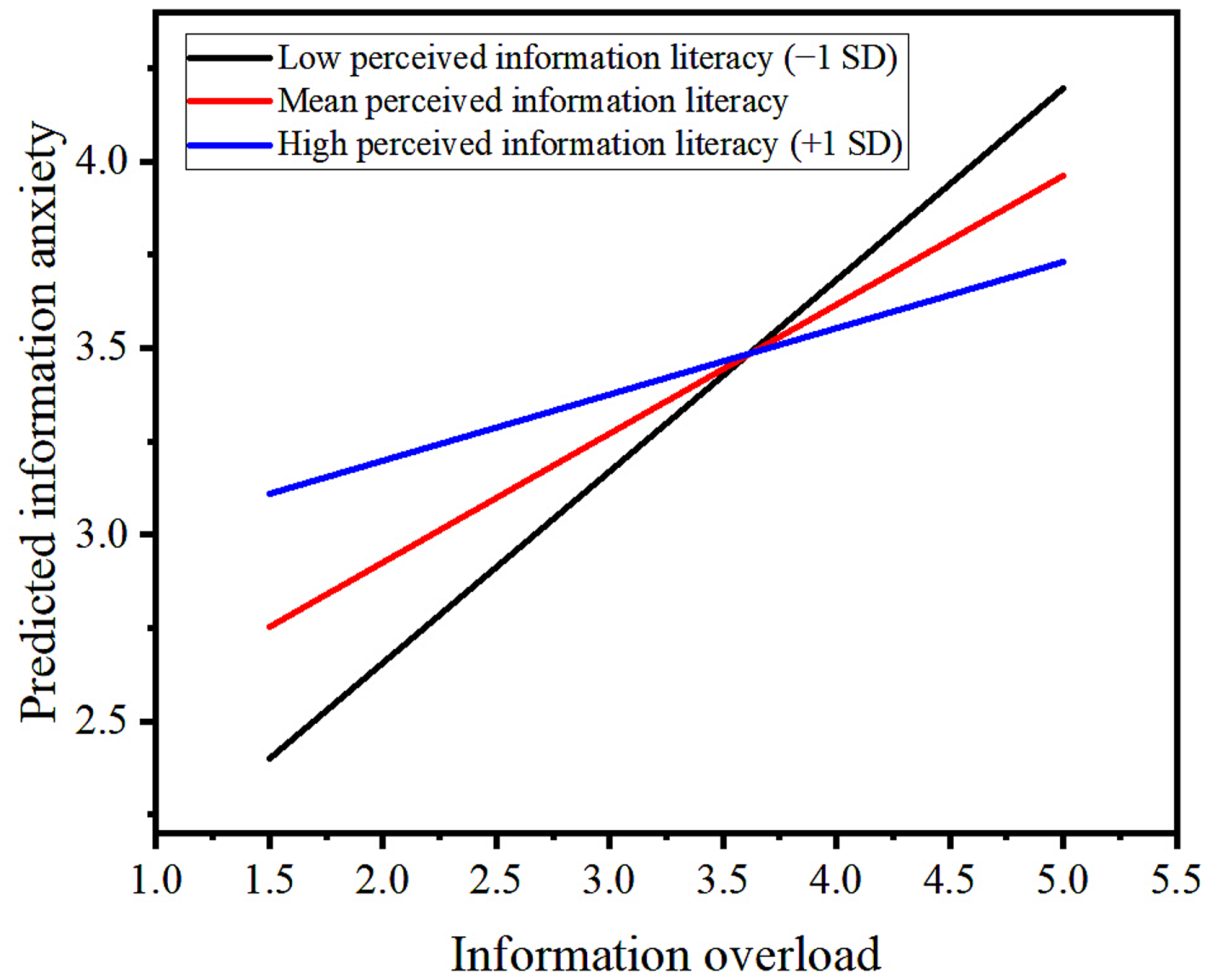
Conditional association between information overload and information anxiety at low, mean, and high levels of perceived information literacy. Covariates were held at their sample means.

#### 3.7.2 Learning self-efficacy

The interaction between information anxiety and learning self-efficacy was positive and statistically significant, B = 0.318, HC3 SE = 0.035, t = 9.135, with a 95% percentile bootstrap confidence interval of [0.251, 0.386]. Adding the interaction term increased the explained variance in self-regulated learning by 0.041.

At one standard deviation below the mean of learning self-efficacy, the conditional association between information anxiety and self-regulated learning was negative and statistically significant, B = −0.482, HC3 SE = 0.040, 95% CI [−0.560, −0.404], *p* < 0.001. At the mean of learning self-efficacy, the association remained negative and statistically significant, B = −0.266, HC3 SE = 0.030, 95% CI [−0.325, −0.207], *p* < 0.001. At one standard deviation above the mean, the association was substantially weaker and was not statistically significant, B = −0.050, HC3 SE = 0.037, 95% CI [−0.122, 0.023], *p* = 0.178.

The Johnson-Neyman analysis identified two threshold values on the observed learning self-efficacy scale, 4.235 and 4.748. The conditional association between information anxiety and self-regulated learning was statistically negative below 4.235, was not statistically significant between 4.235 and 4.748, and was statistically positive above 4.748. In the analytical sample, 59 participants, representing 7.0% of the sample, had learning self-efficacy scores above the upper threshold of 4.748. The positive association therefore represents an upper-tail boundary pattern involving a relatively small portion of the observed learning self-efficacy distribution.

The conditional pattern is illustrated in Figure 4. The interaction coefficient and conditional associations are summarized in Table 7, whereas the Johnson-Neyman threshold results are reported in Supplementary Table S8.

**Figure 4:**
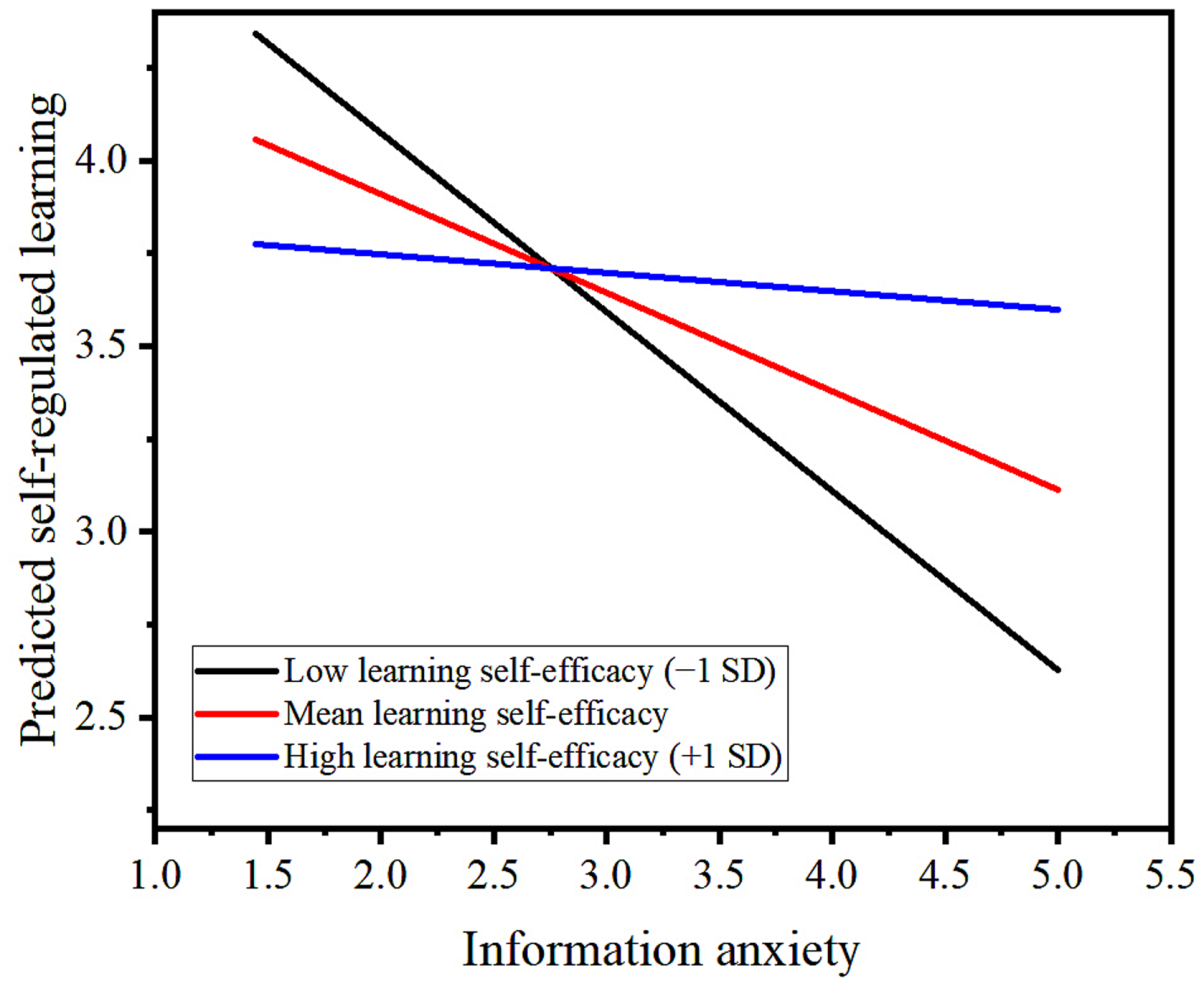
Conditional association between information anxiety and self-regulated learning at low, mean, and high levels of learning self-efficacy. Covariates were held at their sample means.

**Table 7:** Moderation and conditional associations.

Model	Interaction	B	HC3 SE	t	95% bootstrap CI	ΔR^2^
(A) Interaction terms						
IA as outcome	IO×IL	−0.251	0.032	−7.713	[−0.315, −0.190]	0.034
SRL as outcome	IA × LSE	0.318	0.035	9.135	[0.251, 0.386]	0.041

Focal association	Moderator level	Moderator value	Conditional B	HC3 SE	95% CI	*p*
(B) Conditional associations						
IO→IA	Low IL, one SD below mean	2.921	0.513	0.039	[0.436, 0.589]	<0.001
	Mean IL	3.59	0.345	0.03	[0.286, 0.404]	<0.001
	High IL, one SD above mean	4.259	0.178	0.035	[0.109, 0.246]	<0.001
IA → SRL	Low LSE, one SD below mean	2.937	−0.482	0.04	[−0.560, −0.404]	<0.001
	Mean LSE	3.618	−0.266	0.03	[−0.325, −0.207]	<0.001
	High LSE, one SD above mean	4.299	−0.050	0.037	[−0.122, 0.023]	0.178

The model predicting information anxiety controlled for PAI and PC. The model predicting self-regulated learning controlled for PAI, IO, and PC.

### 3.8 Measurement invariance and academic-stage comparisons

The configural model showed acceptable fit across undergraduate, master’s, and doctoral groups, χ^2^(3945) = 4340.379, CFI = 0.991, TLI = 0.991, and RMSEA = 0.011.

Constraining factor loadings to equality did not significantly worsen model fit, Δχ^2^(96) = 110.842, *p* = 0.143, ΔCFI = 0.000, and ΔRMSEA = 0.000. Metric invariance was therefore supported.

Constraining the path from information overload to information anxiety to equality across the groups significantly worsened model fit, Δχ^2^(2) = 16.371, *p* < 0.001. The standardized coefficient was 0.240 for undergraduate students, 0.480 for master’s students, and 0.392 for doctoral students.

Constraining the path from information anxiety to self-regulated learning to equality did not worsen model fit, Δχ^2^(2) = 1.759, *p* = 0.415. Constraining the path from perceived control to self-regulated learning to equality was also nonsignificant, Δχ^2^(2) = 0.416, *p* = 0.812.

Academic-stage heterogeneity was therefore concentrated in the association between information overload and information anxiety among the three formally compared structural paths. Measurement-invariance results and structural comparisons are presented in Table 8. Full group-specific estimates are reported in Supplementary Table S9.

**Table 8:** Measurement invariance and academic-stage structural comparisons.

Model	χ^2^	df	CFI	TLI	RMSEA	Δχ^2^	Δdf	*p*	ΔCFI
(A) Measurement invariance									
Configural	4,340.379	3,945	0.991	0.991	0.011	—	—	—	—
Metric	4,451.221	4,041	0.991	0.991	0.011	110.842	96	0.143	0

Constrained path	Undergraduate β	Master’s β	Doctoral β	Δχ^2^(2)	*p*	Conclusion
(B) Structural path comparisons						
IO→IA	0.24	0.48	0.392	16.371	<0.001	Different across stages
IA → SRL	−0.241	−0.343	−0.268	1.759	0.415	No significant difference
PC → SRL	0.524	0.507	0.48	0.416	0.812	No significant difference

## 4 Discussion

### 4.1 Competing associations in a WeChat-centered social media learning environment

The findings indicate that platform-based academic interaction was associated with self-regulated learning in more than one direction. Its direct association with self-regulated learning was positive, whereas the combined indirect associations involving information overload, perceived control, and information anxiety were negative and larger in absolute magnitude. The estimated total association was therefore negative. The two directions correspond to different components of platform-based academic interaction. The positive direct association is consistent with the learning functions of resource access, coordination, communication, feedback, and help seeking. The negative indirect associations reflect the information-processing demands, reduced manageability, and information-related tension that can accompany the same communication activity. Their coexistence provides the empirical basis for the competing-association pattern examined in this study. This pattern is consistent with research showing that digital interaction can facilitate information retrieval, social exchange, resource acquisition, and learning coordination, while intensive digital communication may also be associated with fatigue, cognitive depletion, and difficulties in managing learning-related information (Cai and Yang, 2025; Wang M. et al., 2025; Wang X. et al., 2025).

The WeChat-centered context helps situate these concurrent associations. Most participants identified WeChat as their primary academic communication platform, and the descriptive context indicators showed that continuous connectivity, fragmented information sharing, responsiveness expectations, and concern about missing academic updates were common features of their communication environment. These indicators were not included as predictors in the structural model and therefore cannot explain the observed associations directly. They nevertheless show that platform-based academic interaction occurred within a setting in which academic resources and information-management demands were closely intertwined.

Recent research on the coordinated use of generative AI and social media has similarly distinguished favorable and unfavorable patterns of digital learning and has identified perceived support as a relevant boundary condition (Chen et al., 2026). The present study extends this line of inquiry by focusing on self-regulated learning and by examining information overload, perceived control, and information anxiety within one specified model. The academic-interaction items referred broadly to digital platforms, while WeChat constituted the dominant communication environment reported by participants. The findings therefore characterize platform-based academic interaction situated within a WeChat-dominant context and direct attention toward the organization and manageability of academic communication.

### 4.2 Information overload, perceived control, and self-regulated learning

The largest specific indirect association followed the specified sequence from platform-based academic interaction to information overload, perceived control, and self-regulated learning. Within the model, information overload was associated with lower perceived control, and perceived control showed the strongest positive association with self-regulated learning. This pattern is consistent with recent evidence connecting information and social overload with cognitive depletion and academic functioning (Wang X. et al., 2025). It also suggests that the relevance of overload may extend beyond the amount of information received to students’ appraisal of whether that information remains manageable.

Cognitive load theory helps explain why these information-management demands are relevant to learning. Message filtering, reconstruction of fragmented information, and repeated integration of information across communication streams consume limited processing resources (Paas et al., 2003; Sweller et al., 2019; Sweller, 2020). Transitions between communication and learning tasks can add further demands because task switching involves measurable performance costs (Monsell, 2003). These processes extend the interpretation of information overload beyond information quantity to the processing required to select, integrate, and prioritize academic information.

Perceived control in the present study differs from general self-control. It refers to students’ reported capacity to decide when to process academic messages, identify priorities, limit disruption, and maintain a learning rhythm. The direct association between platform-based academic interaction and perceived control was small, whereas the association between information overload and perceived control was substantially larger. After information overload was considered, the residual association between platform-based academic interaction and perceived control was small. Lower perceived control was more closely related to the experience of information as excessive, fragmented, repetitive, or difficult to filter. The strong association between perceived control and self-regulated learning is compatible with control-value theory and with research linking regulatory appraisals, academic emotions, and engagement (Shi and Li, 2026). The cross-sectional design leaves the temporal relationship between perceived control and learning regulation open. Longitudinal research can examine whether changes in perceived control precede changes in learning regulation and whether reciprocal relationships develop over time.

### 4.3 Information anxiety as a provisional integrated representation

The measurement analysis identified a single broad factor as the admissible representation of information anxiety in the present sample. Its dimensional interpretation remains provisional. The correlated six-factor model and the second-order model produced inadmissible parameter estimates, even though their global fit indices appeared strong. Concerns about retrieval, quality, processing, security, dependence, and missing information represent different aspects of academic information use. Their high empirical overlap in the present sample also indicates a strong common component of uncertainty and tension associated with difficulties in managing academic information.

Information overload, perceived control, and information anxiety also remained empirically distinguishable. Information overload represented perceived informational demand, perceived control represented appraisal of manageability, and information anxiety represented cognitive and emotional strain. Information anxiety retained a negative association with self-regulated learning after information overload and perceived control were included in the model. This finding is consistent with research indicating that fear of missing information and anxiety related to online communication are associated with students’ attention and academic functioning (Wu et al., 2025). The present construct is more specifically concerned with information needed for academic tasks.

The supplementary item-level diagnostics provide further evidence of substantial similarity within the 18-item measure. Corrected item-total correlations were uniformly high, while deletion of individual items produced little change in the full-scale alpha of 0.979. These results indicate a strong common component across the items and identify item redundancy as an important issue for subsequent psychometric refinement.

The reduced-item sensitivity analyses help separate the stability of the substantive relationships from refinement of the measurement instrument. The six-item domain-balanced score retained high internal consistency, α = 0.940, and the twelve-item score yielded α = 0.968. Both specifications reproduced the principal relationships involving information overload, perceived control, information anxiety, and self-regulated learning, together with the two moderation patterns. The convergence of these results across the full and reduced item sets indicates that the principal observed-score associations were stable across alternative representations of information anxiety.

Further scale development can therefore focus on item refinement and dimensional structure. Independent samples will be important for formal item selection, comparison of bifactor and exploratory structural equation specifications, and evaluation of whether revised items yield clearer differentiation among the six content domains.

### 4.4 Conditional associations involving perceived information literacy and learning self-efficacy

The two moderation analyses identified different conditional relationships within the specified model. At higher levels of perceived information literacy, the positive association between information overload and information anxiety was weaker. Students who reported greater confidence in evaluating relevance, comparing sources, verifying information, and organizing academic materials showed a less pronounced association between information overload and information anxiety. This pattern is consistent with recent research relating digital literacy to learning adaptation, self-efficacy, digital learning, and online self-regulated learning (Chen, 2025; Yuan et al., 2025).

The moderation pattern indicates progressive attenuation of the positive association between information overload and information anxiety as perceived information literacy increases. At one standard deviation above the mean, the association remained positive and statistically significant, although it was substantially weaker than at the mean or at one standard deviation below the mean. The association became non-significant when perceived information literacy exceeded approximately 4.635. Perceived information literacy therefore appears to function as a conditional resource associated with reduced sensitivity of information anxiety to information overload.

Learning self-efficacy was also associated with variation in the relationship between information anxiety and self-regulated learning. The negative association was strongest at low levels of learning self-efficacy, was smaller at the mean, and was not statistically significant at one standard deviation above the mean. This pattern is consistent with research linking self-efficacy with persistence, metacognitive strategy use, and self-regulated learning (Xie, 2026).

The Johnson-Neyman analysis indicated that the conditional association became statistically positive when learning self-efficacy exceeded 4.748. This region represented 59 participants, or 7.0% of the analytical sample. The positive coefficient therefore represents an upper-tail boundary pattern involving relatively few observations. Its substantive meaning remains uncertain because the pattern may reflect characteristics of highly self-efficacious students, a ceiling-related feature of the scale, unmodeled nonlinearity, or sampling variation at the upper end of the distribution. Replication with stronger representation of this score range is required before a substantive interpretation is assigned to the positive region.

The two moderation results correspond to different relationships in the hypothesized model. Perceived information literacy was associated with the strength of the relationship between information overload and information anxiety, whereas learning self-efficacy was associated with the strength of the relationship between information anxiety and self-regulated learning. Their locations in the specified model provide a useful conceptual distinction. The cross-sectional design leaves the temporal ordering of these conditional relationships for longitudinal investigation.

Both interaction patterns remained statistically significant after adjustment for institution membership and under the six-item and twelve-item information-anxiety sensitivity specifications, indicating stability across the supplementary analyses.

### 4.5 Academic-stage and contextual boundaries

The path from information overload to information anxiety was the only examined structural relationship that differed significantly across academic stages. The association was strongest among master’s students, followed by doctoral and undergraduate students. The stronger association among master’s students identifies an exploratory stage-specific pattern. Master’s study commonly combines advanced coursework, developing research responsibilities, thesis preparation, and career-related information demands. Future research can measure these demands directly to determine whether they account for the stronger association between information overload and information anxiety.

The paths from information anxiety to self-regulated learning and from perceived control to self-regulated learning did not differ significantly across the three academic stages. Perceived control was positively associated with self-regulated learning in each group, and information anxiety was negatively associated with self-regulated learning in each group. These results indicate greater cross-stage consistency in the two relationships than in the association between information overload and information anxiety.

The communication context also limits generalization. WeChat groups can differ in instructor presence, peer authority, information accuracy, and expectations of responsiveness (Tan and Ren, 2025). The present findings describe a sample in which WeChat was the dominant academic communication platform, but the measures did not distinguish among teacher-managed groups, student-organized groups, private conversations, and other platform channels. The pattern may differ in learning environments where academic communication is concentrated in formal learning-management systems, email, or institutionally separated channels.

Institutional context represents an additional boundary. The supplementary clustering diagnostics indicated little between-institution variance in the present sample, while the six participating institutions provide a limited basis for broader institution-level inference.

### 4.6 Theoretical implications

The study contributes to research on self-regulated learning by highlighting the management of academic information as a relevant contextual condition. Self-regulated learning continues to concern goal setting, planning, monitoring, attention, effort, and strategy adjustment. The present findings suggest that students’ reported regulation of these activities is also associated with whether the surrounding information environment is experienced as manageable. This extends the contextual analysis of self-regulated learning by situating information manageability as a condition surrounding the enactment of goal setting, attention, monitoring, effort, and strategy adjustment in social media environments.

The findings also clarify the meaning of competing associations in social media learning. Platform-based academic interaction was positively associated with self-regulated learning after the other variables were considered, while its indirect associations involving information overload, perceived control, and information anxiety were negative. Perceived control emerged as a prominent correlate linking informational demand, emotional strain, and learning regulation within the specified model. The functional differentiation among information overload, perceived control, and information anxiety provides a further contribution. Information overload captures information-processing demand, perceived control captures appraisal of manageability, and information anxiety captures the cognitive and emotional response associated with difficulties in governing academic information. Their joint analysis connects resource demands, regulatory appraisal, and emotional experience within the same social media learning context. The moderation results further indicate that perceived information literacy and learning self-efficacy were related to the strength of different associations.

Perceived information literacy was associated with variation in the overload to anxiety relationship, whereas learning self-efficacy was associated with variation in the anxiety to self-regulated learning relationship. This pattern suggests that perceived information-management capability and perceived learning capability correspond with different relationships within the proposed model.

These contributions concern the organization of relationships within a cross-sectional theoretical model. Longitudinal evidence can extend this framework by examining the temporal development of the identified relationships.

### 4.7 Practical implications

The results suggest that practical attention may be directed toward both communication design and students’ information-management capacities. At the environmental level, instructors and institutions may benefit from identifying a clear channel for high-priority notices, distinguishing urgent from non-urgent information, summarizing decisions and deadlines, reducing repeated forwarding, and limiting context-free screenshots. Clear expectations concerning response time may also reduce the perceived requirement for continuous monitoring.

At the learner level, educational support may include the evaluation of information quality, identification of task-relevant messages, comparison of sources, organization and archiving of academic materials, and planned rather than continuous checking of platform messages. Support for learning self-efficacy may additionally emphasize persistence, restoration of attention after interruption, and adjustment of learning plans following setbacks. Individual-level support and responsible communication design are complementary components of an information-manageable learning environment. Excessive redundancy, ambiguity, and responsiveness expectations can continue to generate information demands even among students reporting high perceived capability.

### 4.8 Limitations and future research

The study has several limitations. First, the cross-sectional design limits inference concerning temporal ordering and causality. The direct, indirect, moderated, and multigroup results characterize statistical relationships within the theoretically specified model. Longitudinal or experience-sampling designs could examine the temporal relationships among platform interaction, information overload, perceived control, information anxiety, and self-regulated learning.

Second, all principal constructs were measured through self-report in the same survey. Response style, common method variance, and subjective interpretation may have contributed to the observed associations. Harman’s single-factor analysis provides only a limited diagnostic of common method variance. Future research should combine questionnaires with platform-use records, message characteristics, task-performance indicators, and performance-based assessments of information literacy.

Third, the non-probability recruitment strategy limits population-level representativeness. The inclusion of six universities from different regions and institutional types increased sample heterogeneity, while broader generalization to Chinese university students requires more systematic sampling designs.

Fourth, students were nested within six institutions. The clustering diagnostics indicated very small institution-level variance, and the principal composite-score relationships remained stable after adjustment for institution membership. The limited number of institutional clusters restricts the precision of institution-level inference. Studies involving a larger number of universities can provide stronger tests of contextual variation.

Fifth, the information-anxiety measure requires independent psychometric development. The very high reliability coefficient, narrow factor-loading range, and uniformly high corrected item-total correlations indicate substantial commonality among the current items. These measurement characteristics co-occurred with unusually close fit in both the measurement and structural models, making independent replication important for evaluating whether the observed loading patterns and model-fit characteristics reproduce in new samples. The six-item and twelve-item sensitivity specifications reproduced the principal observed-score relationships, which supports the robustness of those relationships across alternative item sets. Future measurement research can extend the present qualitative expert review through multi-expert content-validity assessment, apply formal item-reduction procedures, revise item content, compare bifactor and exploratory structural equation specifications, and evaluate the resulting measures in independent samples.

Sixth, platform-based academic interaction was measured broadly. The sample’s platform-use profile and contextual indicators support the WeChat-centered interpretation, while the interaction scale itself assessed platform-based academic interaction across digital communication channels. Future studies could distinguish interactions in teacher-managed WeChat groups, student-organized groups, private communication, formal learning-management systems, and emerging communication environments involving social media and generative AI.

Finally, the doctoral subgroup was smaller than the undergraduate and master’s groups, and the positive conditional association observed at the extreme upper end of learning self-efficacy was based on a limited portion of the sample. Both findings require replication before stronger developmental or substantive conclusions are drawn.

## 5 Conclusion

Platform-based academic interaction in a WeChat-centered social media learning environment showed both positive and negative associations with self-regulated learning. Its direct association with self-regulated learning was positive, while the estimated indirect associations involving information overload, perceived control, and information anxiety were negative and greater in absolute magnitude, yielding a negative estimated total association. Within the specified model, perceived control emerged as a prominent correlate of self-regulated learning, and the largest specific indirect association involved platform-based academic interaction, information overload, perceived control, and self-regulated learning.

The association between information overload and information anxiety was weaker at higher levels of perceived information literacy, while the negative association between information anxiety and self-regulated learning was weaker at higher levels of learning self-efficacy. The path from information overload to information anxiety differed across academic stages and was strongest among master’s students, whereas the other two paths examined across stages did not differ significantly. These findings highlight the relevance of both academic communication design and students’ perceived information-management and learning capacities. The supplementary institutional and reduced-item analyses further showed that the principal observed-score relationships remained stable across the additional sensitivity specifications. The findings therefore characterize cross-sectional associations situated within a WeChat-dominant communication environment.

## Data Availability

The raw data supporting the conclusions of this article will be made available by the authors, without undue reservation.
