# Supplementary material for: Platform-based academic interaction and self-regulated learning in a WeChat-centered social media environment: information overload, perceived control, and information anxiety
Source: Front Psychol. 2026 Sep 21;17:1893472. doi: 10.3389/fpsyg.2026.1893472 (PMC13635151; doi:10.3389/fpsyg.2026.1893472)
Supplement: Supplementary file 1 [file Supplementary_file_1.docx]

Supplementary Material

## Supplementary Table S1. Main measurement items

All items were rated on a five-point Likert scale ranging from 1 = strongly disagree to 5 = strongly agree.

**(A)** Platform-based academic interaction

| **Code** | **Content domain** | **Item wording** |
| --- | --- | --- |
| PAI1 | Course communication | I receive course-related information through digital communication platforms. |
| PAI2 | Course communication | I keep track of course requirements through platform-based messages. |
| PAI3 | Course communication | I use digital platforms to communicate with teachers or teaching assistants about academic matters. |
| PAI4 | Course communication | I regularly receive platform-based course messages that are relevant to my learning activities. |
| PAI5 | Peer collaboration | I use digital platforms to discuss academic tasks with classmates or peers. |
| PAI6 | Peer collaboration | I coordinate group learning or project work through platform-based peer communication. |
| PAI7 | Peer collaboration | I exchange learning resources with peers through digital platforms. |
| PAI8 | Peer collaboration | I participate in academic discussions with peers through digital platforms. |
| PAI9 | Information acquisition | I obtain learning materials or academic information through digital platforms. |
| PAI10 | Information acquisition | I follow academic notices, lectures, competitions, or learning opportunities through digital platforms. |
| PAI11 | Information acquisition | I receive platform-based information that is relevant to my learning priorities. |
| PAI12 | Information acquisition | Digital platforms are an important channel for acquiring academic information in my daily learning. |

*Note.* Developed with reference to peer-reviewed research on social media supported learning and digital academic communication (Greenhow and Lewin, 2016; Greenhow and Galvin, 2020; Manca, 2020; Barrot, 2022).

**(B)** Information overload

| **Code** | **Content domain** | **Item wording** |
| --- | --- | --- |
| IO1 | Quantity | The amount of learning-related information I receive through digital platforms often exceeds my capacity. |
| IO2 | Speed | Learning-related messages arrive so quickly that I find it difficult to process them in time. |
| IO3 | Fragmentation | Academic information on platforms is often fragmented and difficult to integrate. |
| IO4 | Filtering difficulty | I need to spend considerable effort filtering useful learning information from irrelevant messages. |
| IO5 | Quantity | I often feel that there is too much platform-based academic information to handle effectively. |
| IO6 | Redundancy | Repeated or overlapping academic messages make it difficult for me to identify what is important. |

## Note. Developed with reference to Eppler and Mengis (2004), Karr-Wisniewski and Lu (2010), and Pang (2021).

**(C)** Perceived control

| **Code** | **Content domain** | **Item wording** |
| --- | --- | --- |
| PC1 | Timing control | I can decide when and how to deal with platform-based academic information. |
| PC2 | Disruption control | I feel able to keep irrelevant platform messages from disrupting my learning plan. |
| PC3 | Regulatory appraisal | I feel that platform-based academic information remains manageable for my learning priorities. |
| PC4 | Learning rhythm | I can maintain my own learning rhythm despite frequent academic messages. |
| PC5 | Overall control | I feel able to manage platform-based information without losing control of my study schedule. |

## *Note.* Developed with reference to peer-reviewed research on control appraisals and achievement emotions (Skinner, 1996; Pekrun, 2006).

**(D)** Information anxiety

| **Code** | **Content domain** | **Item wording** |
| --- | --- | --- |
| IA-R1 | Information retrieval | I worry that I cannot find important learning information when I need it. |
| IA-R2 | Information retrieval | I feel anxious when academic information is scattered across different platform groups or channels. |
| IA-R3 | Information retrieval | I worry that I may not be able to retrieve previously shared learning materials from platform message histories. |
| IA-Q1 | Information quality | I feel uncertain about whether platform-based academic information is accurate. |
| IA-Q2 | Information quality | I worry that I may rely on learning information that is incomplete or unreliable. |
| IA-Q3 | Information quality | I feel anxious when different platform messages provide inconsistent academic information. |
| IA-P1 | Information processing | I feel tense when I have to make sense of many platform-based academic messages within a short time. |
| IA-P2 | Information processing | I worry that I may misunderstand academic requirements because the information is difficult to process. |
| IA-P3 | Information processing | I feel anxious when I cannot turn scattered platform messages into a clear learning plan. |
| IA-S1 | Information security | I worry about the privacy or security of academic information shared through platforms. |
| IA-S2 | Information security | I feel uneasy when important academic materials are stored or circulated through digital platforms. |
| IA-S3 | Information security | I worry that platform-based academic information may be lost, leaked, or misused. |
| IA-D1 | Dependence | I feel uneasy because my learning depends heavily on digital communication platforms. |
| IA-D2 | Dependence | I feel uneasy when my learning routines depend too much on platform notifications and messages. |
| IA-D3 | Dependence | I feel anxious when I cannot access the platform channels used for academic communication. |
| IA-M1 | Missing information | I worry about missing important learning information. |
| IA-M2 | Missing information | I feel anxious when I have not checked academic platform messages for a while. |
| IA-M3 | Missing information | I worry that others may receive important academic updates earlier than I do. |

*Note.* Items were developed with reference to peer-reviewed research on information anxiety, information overload, fear of missing out, and digital dependence (Bawden and Robinson, 2009; Dhir et al., 2018; Elhai et al., 2020; Wu et al., 2025). The six domains were used to ensure content breadth. All 18 items were modeled as indicators of a single information-anxiety factor. A single-factor representation provided the admissible specification used in the primary analysis, and its dimensional interpretation is treated as provisional pending independent validation.

**(E)** Self-regulated learning

| **Code** | **Content domain** | **Item wording** |
| --- | --- | --- |
| SRL1 | Planning | I set clear learning goals before starting academic tasks. |
| SRL2 | Planning | I make plans for completing learning tasks. |
| SRL3 | Planning | I arrange my study time according to task priorities. |
| SRL4 | Attention | I try to stay focused when working on learning tasks. |
| SRL5 | Attention | I reduce distractions when I need to study. |
| SRL6 | Attention | I can return my attention to learning after being interrupted. |
| SRL7 | Monitoring | I monitor whether my learning progress is consistent with my plan. |
| SRL8 | Monitoring | I check whether I understand the learning materials. |
| SRL9 | Monitoring | I evaluate whether my current learning strategies are effective. |
| SRL10 | Adjustment | I adjust my learning strategies when necessary. |
| SRL11 | Adjustment | I change my study plan when I find that it is not working well. |
| SRL12 | Adjustment | I seek alternative ways to complete learning tasks when difficulties arise. |

*Note.* Adapted from peer-reviewed research on self-regulated learning in online and blended learning environments (Barnard et al., 2009; Broadbent and Poon, 2015; Panadero, 2017).

**(F)** Perceived information literacy

| **Code** | **Content domain** | **Item wording** |
| --- | --- | --- |
| IL1 | Information evaluation | I can judge whether platform-based academic information is reliable. |
| IL2 | Source comparison | I can compare information from different sources before using it for learning. |
| IL3 | Relevance judgment | I can identify which platform messages are relevant to my learning goals. |
| IL4 | Information organization | I can categorize and save platform-based academic information for later use. |
| IL5 | Information verification | I can verify important learning information before acting on it. |

*Note.* Developed with reference to peer-reviewed research on information-literacy self-efficacy and perceived capacity to evaluate, verify, select, and manage information (Kurbanoglu et al., 2006; Aharony and Gazit, 2020; Shonfeld et al., 2022).

**(G)** Learning self-efficacy

| **Code** | **Content domain** | **Item wording** |
| --- | --- | --- |
| LSE1 | Confidence | I am confident that I can complete my learning tasks even when they are difficult. |
| LSE2 | Persistence | I can persist in learning when I encounter obstacles. |
| LSE3 | Recovery belief | I believe I can regain confidence in learning after disruptions or setbacks. |
| LSE4 | Challenge management | I believe I can manage academic challenges effectively. |
| LSE5 | Persistence under stress | Even when I feel stressed, I can continue working toward my learning goals. |

*Note.* Developed with reference to peer-reviewed research on self-efficacy, academic self-efficacy, and self-regulated learning (Bandura, 1977; Zimmerman and Bandura, 1994; Zimmerman, 2000; Honicke and Broadbent, 2016; Schunk and DiBenedetto, 2020).

## Supplementary Table S2. WeChat-centered social media context

**(A)** Institutional and platform context represented in the analytical file

| **Context variable** | **Category** | **n** | **%** |
| --- | --- | --- | --- |
| Anonymous institution | U01 | 148 | 17.5 |
|  | U02 | 136 | 16.1 |
|  | U03 | 142 | 16.8 |
|  | U04 | 147 | 17.4 |
|  | U05 | 139 | 16.4 |
|  | U06 | 134 | 15.8 |
| Region | Eastern China | 284 | 33.6 |
|  | Central China | 289 | 34.2 |
|  | Western China | 273 | 32.3 |
| Institution type | Comprehensive university | 295 | 34.9 |
|  | Science and engineering university | 275 | 32.5 |
|  | Teacher education university | 276 | 32.6 |
| Primary academic platform | WeChat | 776 | 91.7 |
|  | QQ | 37 | 4.4 |
|  | DingTalk | 18 | 2.1 |
|  | LMS or other | 15 | 1.8 |
| WeChat academic use | Yes | 833 | 98.5 |
|  | No | 13 | 1.5 |

**(B)** Social media context indicators

| **Variable** | **Context represented** | **M** | **SD** | **4 or 5 (%)** |
| --- | --- | --- | --- | --- |
| SMC1 | Continuous connectivity of academic communication | 3.79 | 0.846 | 64.3 |
| SMC2 | Overlap between academic and personal communication | 3.534 | 0.939 | 51.4 |
| SMC3 | Rapid and fragmented information sharing | 3.831 | 0.856 | 65.6 |
| SMC4 | Expectation of timely responsiveness | 3.68 | 0.889 | 59.2 |
| SMC5 | Concern about quality or reliability of shared academic information | 3.382 | 0.854 | 45.6 |
| SMC6 | Pressure associated with missing important academic updates | 3.755 | 0.912 | 62.8 |

## *Note.* The six social media context indicators are reported descriptively as heterogeneous contextual characteristics, and each indicator is interpreted separately.

**Supplementary Table S3.** Item-level standardized factor loadings and construct-level measurement properties

| **Construct** | **Item** | **Standardized loading** | **Cronbach’s α** | **CR** | **AVE** |
| --- | --- | --- | --- | --- | --- |
| PAI | PAI1 | 0.844 | 0.968 | 0.968 | 0.716 |
|  | PAI2 | 0.853 |  |  |  |
|  | PAI3 | 0.848 |  |  |  |
|  | PAI4 | 0.853 |  |  |  |
|  | PAI5 | 0.838 |  |  |  |
|  | PAI6 | 0.85 |  |  |  |
|  | PAI7 | 0.843 |  |  |  |
|  | PAI8 | 0.855 |  |  |  |
|  | PAI9 | 0.851 |  |  |  |
|  | PAI10 | 0.836 |  |  |  |
|  | PAI11 | 0.842 |  |  |  |
|  | PAI12 | 0.844 |  |  |  |
| IO | IO1 | 0.852 | 0.942 | 0.942 | 0.73 |
|  | IO2 | 0.835 |  |  |  |
|  | IO3 | 0.866 |  |  |  |
|  | IO4 | 0.84 |  |  |  |
|  | IO5 | 0.867 |  |  |  |
|  | IO6 | 0.866 |  |  |  |
| PC | PC1 | 0.882 | 0.938 | 0.938 | 0.753 |
|  | PC2 | 0.865 |  |  |  |
|  | PC3 | 0.866 |  |  |  |
|  | PC4 | 0.862 |  |  |  |
|  | PC5 | 0.863 |  |  |  |
| IA | IA-R1 | 0.844 | 0.979 | 0.979 | 0.717 |
|  | IA-R2 | 0.851 |  |  |  |
|  | IA-R3 | 0.854 |  |  |  |
|  | IA-Q1 | 0.838 |  |  |  |
|  | IA-Q2 | 0.845 |  |  |  |
|  | IA-Q3 | 0.848 |  |  |  |
|  | IA-P1 | 0.847 |  |  |  |
|  | IA-P2 | 0.839 |  |  |  |
|  | IA-P3 | 0.855 |  |  |  |
|  | IA-S1 | 0.835 |  |  |  |
|  | IA-S2 | 0.854 |  |  |  |
|  | IA-S3 | 0.852 |  |  |  |
|  | IA-D1 | 0.844 |  |  |  |
|  | IA-D2 | 0.838 |  |  |  |
|  | IA-D3 | 0.853 |  |  |  |
|  | IA-M1 | 0.843 |  |  |  |
|  | IA-M2 | 0.863 |  |  |  |
|  | IA-M3 | 0.843 |  |  |  |
| SRL | SRL1 | 0.854 | 0.969 | 0.969 | 0.725 |
|  | SRL2 | 0.86 |  |  |  |
|  | SRL3 | 0.83 |  |  |  |
|  | SRL4 | 0.854 |  |  |  |
|  | SRL5 | 0.848 |  |  |  |
|  | SRL6 | 0.857 |  |  |  |
|  | SRL7 | 0.841 |  |  |  |
|  | SRL8 | 0.867 |  |  |  |
|  | SRL9 | 0.851 |  |  |  |
|  | SRL10 | 0.85 |  |  |  |
|  | SRL11 | 0.837 |  |  |  |
|  | SRL12 | 0.869 |  |  |  |
| IL | IL1 | 0.823 | 0.923 | 0.923 | 0.706 |
|  | IL2 | 0.844 |  |  |  |
|  | IL3 | 0.865 |  |  |  |
|  | IL4 | 0.832 |  |  |  |
|  | IL5 | 0.837 |  |  |  |
| LSE | LSE1 | 0.86 | 0.929 | 0.929 | 0.723 |
|  | LSE2 | 0.85 |  |  |  |
|  | LSE3 | 0.844 |  |  |  |
|  | LSE4 | 0.856 |  |  |  |
|  | LSE5 | 0.84 |  |  |  |

*Note.* All standardized factor loadings were statistically significant at p<.001. Cronbach’s α, composite reliability (CR), and average variance extracted (AVE) are reported on the first row for each construct.

## Supplementary Table S4. HTMT matrix

| **Construct** | **PAI** | **IO** | **PC** | **IA** | **SRL** | **IL** | **LSE** |
| --- | --- | --- | --- | --- | --- | --- | --- |
| PAI | — |  |  |  |  |  |  |
| IO | 0.5 | — |  |  |  |  |  |
| PC | 0.322 | 0.524 | — |  |  |  |  |
| IA | 0.462 | 0.626 | 0.576 | — |  |  |  |
| SRL | 0.142 | 0.497 | 0.688 | 0.578 | — |  |  |
| IL | 0.134 | 0.183 | 0.408 | 0.173 | 0.503 | — |  |
| LSE | 0.097 | 0.206 | 0.448 | 0.237 | 0.531 | 0.42 | — |

*Note.* All HTMT values were below .700.

## Supplementary Table S5. Diagnostic and robustness checks

**(A)** Harman’s single-factor analysis and collinearity

| **Diagnostic** | **Result** |
| --- | --- |
| First unrotated factor | 39.24% of total variance |
| VIF in equation predicting IO | 1 |
| VIF in equation predicting PC | Approximately 1.29 |
| VIF in equation predicting IA | Approximately 1.31 to 1.56 |
| VIF in equation predicting SRL | Approximately 1.37 to 1.90 |
| VIF in moderation model predicting IA | 1.00 to 1.58 |
| VIF in moderation model predicting SRL | 1.02 to 1.92 |

**(B)** Moderation-model diagnostics

| **Diagnostic** | **Model predicting IA** | **Model predicting SRL** |
| --- | --- | --- |
| Breusch-Pagan p | 0.216 | 0.146 |
| White test p | 0.576 | 0.461 |
| Maximum Cook’s distance | 0.034 | 0.056 |
| Cases with absolute standardized residual greater than 3 | 0 | 1 |
| Interaction significant with HC3 SE | Yes | Yes |
| Interaction significant with bootstrap CI | Yes | Yes |

**(C)** Institutional clustering diagnostics

| **Construct** | **Random-intercept variance** | **Residual variance** | **ICC** |
| --- | --- | --- | --- |
| PAI | 0.000000 | 0.435860 | .000 |
| IO | 0.000465 | 0.488127 | .001 |
| PC | 0.001653 | 0.548746 | .003 |
| IA | 0.000000 | 0.440720 | .000 |
| SRL | 0.000000 | 0.463505 | .000 |
| IL | 0.000000 | 0.447049 | .000 |
| LSE | 0.003569 | 0.460497 | .008 |

*Note.* Variance components were estimated from empty random-intercept models with institution as the clustering unit. Random-intercept variance estimates at zero corresponded to boundary solutions. With six participating institutions, the ICCs are used as descriptive clustering diagnostics.

## Supplementary Table S6. Sensitivity analyses

**(A)** Core paths with demographic and institution controls

| **Path** | **Baseline β** | **Controlled β** | **Controlled p** |
| --- | --- | --- | --- |
| PAI→IO | 0.477 | 0.476 | <.001 |
| PAI→PC | −.092 | −.093 | 0.007 |
| IO→PC | −.449 | −.458 | <.001 |
| PAI→IA | 0.181 | 0.179 | <.001 |
| IO→IA | 0.356 | 0.365 | <.001 |
| PC→IA | −.322 | −.320 | <.001 |
| PAI→SRL | 0.223 | 0.222 | <.001 |
| IO→SRL | −.167 | −.176 | <.001 |
| PC→SRL | 0.476 | 0.482 | <.001 |
| IA→SRL | −.300 | −.285 | <.001 |

**(B)** Sensitivity analyses in the WeChat subsamples

| **Path** | **Full sample N=846** | **WeChat academic users N=833** | **Primary WeChat N=776** |
| --- | --- | --- | --- |
| PAI→IO | .477*** | .480*** | .484*** |
| PAI→PC | −.092** | −.097** | −.104** |
| IO→PC | −.449*** | −.448*** | −.439*** |
| PAI→IA | .181*** | .178*** | .172*** |
| IO→IA | .356*** | .359*** | .357*** |
| PC→IA | −.322*** | −.319*** | −.328*** |
| PAI→SRL | .223*** | .223*** | .204*** |
| IO→SRL | −.167*** | −.162*** | −.151*** |
| PC→SRL | .476*** | .480*** | .475*** |
| IA→SRL | −.300*** | −.300*** | −.308*** |

**(C)** Moderation models with demographic and institution controls

| **Interaction** | **Baseline B** | **Controlled B** | **HC3 SE** | **95% CI** | **p** |
| --- | --- | --- | --- | --- | --- |
| IO×IL predicting IA | −.251 | −.240 | 0.033 | [−.305, −.174] | <.001 |
| IA×LSE predicting SRL | 0.318 | 0.317 | 0.036 | [.245, .388] | <.001 |

## *Note.* All coefficients in this table are based on composite scale scores. Panels A and B provide sensitivity estimates for the core structural paths, for which the primary latent-variable estimates are reported in Table 5 of the main manuscript. Panel C reports composite-score moderation estimates after adjustment for demographic and institutional variables.

## Supplementary Table S7. Information-anxiety measurement and reduced-item sensitivity analyses

**(A)** Alternative information-anxiety measurement models

| **Model** | **Statistical characteristics** | **Interpretation** |
| --- | --- | --- |
| Single-factor model | Admissible parameter estimates and a positive-definite covariance matrix | Admissible primary representation; dimensional interpretation treated as provisional |
| Six correlated factors | Several latent correlations approached or exceeded 1.00; the latent covariance matrix was not positive definite | Inadmissible; empirical separation among the six domains was insufficient in this sample |
| Second-order model | Negative residual variances for first-order factors and standardized second-order loadings greater than 1.00 | Inadmissible higher-order specification |

**(B)** Item-level diagnostics for the 18-item information-anxiety measure

| **Diagnostic** | **Result** |
| --- | --- |
| Cronbach’s α | .979 |
| Corrected item-total correlation | .825 to .854 |
| Cronbach’s α if item deleted | .9772 to .9775 |

**(C)** Reduced-item sensitivity specifications

| **Specification** | **Selection rule** | **α** | **IO→IA β** | **PC→IA β** | **IA→SRL β** | **IO×IL β** | **IA×LSE β** |
| --- | --- | --- | --- | --- | --- | --- | --- |
| IA6 (6 items) | Second item from each domain | .940 | .351*** | −.306*** | −.285*** | −.174*** | .210*** |
| IA12 (12 items) | First two items from each domain | .968 | .358*** | −.319*** | −.292*** | −.186*** | .203*** |

*Note.* IA6 used IA-R2, IA-Q2, IA-P2, IA-S2, IA-D2, and IA-M2. IA12 used the first two items from each of the six content domains. These domain-balanced scores were created solely as exploratory sensitivity specifications for the observed-score analyses. Formal short-form development requires independent psychometric validation. Coefficients are standardized β values from the corresponding composite-score sensitivity regressions. ***p<.001.

## Supplementary Table S8. Detailed moderation and Johnson-Neyman results

**(A)** Moderation of the association between information overload and information anxiety

| **Result** | **Estimate** |
| --- | --- |
| Interaction B | −.251 |
| OLS SE | 0.033 |
| HC3 SE | 0.032 |
| Bootstrap SE | 0.032 |
| Bootstrap 95% CI | [−.315, −.190] |
| Full-model R² | 0.507 |
| Adjusted R² | 0.504 |
| Interaction ΔR² | 0.034 |
| F change | 57.746 |
| Threshold within observed range | IL=4.635 |
| Observed IL range | 1.60 to 5.00 |

**(B)** Moderation of the association between information anxiety and self-regulated learning

| **Result** | **Estimate** |
| --- | --- |
| Interaction B | 0.318 |
| OLS SE | 0.034 |
| HC3 SE | 0.035 |
| Bootstrap SE | 0.034 |
| Bootstrap 95% CI | [.251, .386] |
| Full-model R² | 0.615 |
| Adjusted R² | 0.612 |
| Interaction ΔR² | 0.041 |
| F change | 88.474 |
| Lower threshold | LSE=4.235 |
| Upper threshold | LSE=4.748 |
| Observed LSE range | 1.80 to 5.00 |
| Participants above the upper threshold | 59 |
| Percentage above the upper threshold | 7.0% |

## *Note.* The conditional association between information anxiety and self-regulated learning was statistically negative below LSE=4.235, was not statistically significant between LSE=4.235 and LSE=4.748, and was statistically positive above LSE=4.748. Scores above the upper threshold corresponded to learning self-efficacy composite scores of 4.8 or 5.0 and included 59 participants, representing 7.0% of the analytical sample. The positive coefficient therefore represents an upper-tail boundary pattern. The model controlled for platform-based academic interaction, information overload, and perceived control.

## Supplementary Table S9. Academic-stage measurement and structural comparisons

**(A)** Measurement invariance

| **Model** | **χ²** | **df** | **χ²/df** | **CFI** | **TLI** | **RMSEA** | **RMR** |
| --- | --- | --- | --- | --- | --- | --- | --- |
| Configural | 4340.379 | 3945 | 1.1 | 0.991 | 0.991 | 0.011 | 0.021 |
| Metric | 4451.221 | 4041 | 1.102 | 0.991 | 0.991 | 0.011 | 0.025 |

Comparison of the metric and configural models: Δχ²(96)=110.842, p=.143; ΔCFI=.000; ΔRMSEA=.000

**(B)** Structural coefficients by academic stage

| **Path** | **Undergraduate β** | **p** | **Master’s β** | **p** | **Doctoral β** | **p** |
| --- | --- | --- | --- | --- | --- | --- |
| PAI→IO | 0.463 | <.001 | 0.508 | <.001 | 0.552 | <.001 |
| PAI→PC | −.081 | 0.16 | −.162 | 0.004 | 0.137 | 0.111 |
| IO→PC | −.459 | <.001 | −.433 | <.001 | −.677 | <.001 |
| PAI→IA | 0.153 | 0.002 | 0.147 | <.001 | 0.232 | 0.003 |
| IO→IA | 0.24 | <.001 | 0.48 | <.001 | 0.392 | <.001 |
| PC→IA | −.453 | <.001 | −.279 | <.001 | −.193 | 0.022 |
| PAI→SRL | 0.233 | <.001 | 0.242 | <.001 | 0.256 | 0.002 |
| IO→SRL | −.193 | <.001 | −.159 | 0.004 | −.136 | 0.196 |
| PC→SRL | 0.524 | <.001 | 0.507 | <.001 | 0.48 | <.001 |
| IA→SRL | −.241 | <.001 | −.343 | <.001 | −.268 | 0.002 |

*Note.* Formal equality tests provide the basis for interpreting between-group differences. Among the three focal structural paths tested, only the path from information overload to information anxiety differed significantly across academic stages.
